# Multiscale computational modeling to quantify how spiral artery remodeling alters wall shear stress on placental villi

**DOI:** 10.1371/journal.pcbi.1014769

**Published:** 2026-09-16

**Authors:** Armita Najmi, Noelia Grande Gutiérrez

**Affiliations:** Department of Mechanical Engineering, Carnegie Mellon University, Pittsburgh, Pennsylvania, United States of America; Islamic Azad University Arsanjan Branch, IRAN, ISLAMIC REPUBLIC OF

## Abstract

Proper placental development is essential for a healthy pregnancy. It depends on the remodeling of the maternal uterine vasculature to meet fetal demands while maintaining physiological intervillous space (IVS) hemodynamics for biochemical exchange. Terminal villi, the primary sites of feto-maternal exchange, exhibit impaired development in pregnancies complicated by intrauterine growth restriction and preeclampsia, which are also associated with incomplete spiral artery (SA) remodeling. Despite this association, the mechanistic link between maternal blood flow and villous development remains unclear. Here, we investigate whether incomplete SA remodeling alters IVS hemodynamics and increases wall shear stress (WSS) on placental villi, potentially impairing terminal villi formation. Computing WSS throughout an entire placentone is challenging due to uncertainty in placental microstructure and the computational cost of resolving microscale hemodynamics. We propose a novel multiscale computational framework to quantify WSS on placental villi at the end of the second trimester, when WSS may affect terminal villi development. A macroscale placentone model is used to compute IVS velocities, which are coupled with microscale models of intermediate villi to estimate villous WSS across physiologically relevant flow conditions. We simulate IVS hemodynamics in healthy pregnancy and varying degrees of incomplete SA remodeling. Our results show that IVS velocity is the primary determinant of mean villous WSS, whereas villous type and orientation have comparatively weaker effects. In healthy placentones, most villi experience a mean WSS of 0.001–1 Pa, with higher stresses localized near the free-of-villi cavity. By correlating these estimates with regions naturally devoid of terminal villi, we identify a mean WSS range of approximately 0.71–1.44 Pa that may inhibit terminal villi formation. Incomplete SA remodeling significantly increases WSS, reaching levels consistent with villous tissue loss and placental lake formation. These findings suggest a mechanistic link between uteroplacental hemodynamics and villi development, establishing physiological shear-stress thresholds relevant to placental health.

## Author summary

Mechanical forces play an essential role in regulating cell behavior by influencing mechanosensory pathways and biochemical signaling. Although biological processes are often studied from a biochemical perspective, mechanical cues can strongly shape tissue development. This is particularly relevant for the placenta, a growing organ whose proper development is essential for a healthy pregnancy. Placental villi are small, tree-like structures that contain fetal blood vessels and facilitate the exchange of oxygen and nutrients between mother and fetus; their impaired development is commonly observed in complicated pregnancies. In many such pregnancies, the maternal spiral arteries that supply blood to the placenta are only partially remodeled. This incomplete remodeling alters placental blood flow and changes the mechanical forces acting on placental villi. In this study, we developed a computational framework to quantify wall shear stress acting on placental villi under healthy and pathological conditions in a computationally efficient manner. Using this approach, we identified a range of shear stress levels that may hinder terminal villi formation. These findings provide new insight into how altered blood flow mechanics may contribute to abnormal placental development and inform future experimental studies on trophoblast mechanobiology.

## Introduction

**Fig 1:**
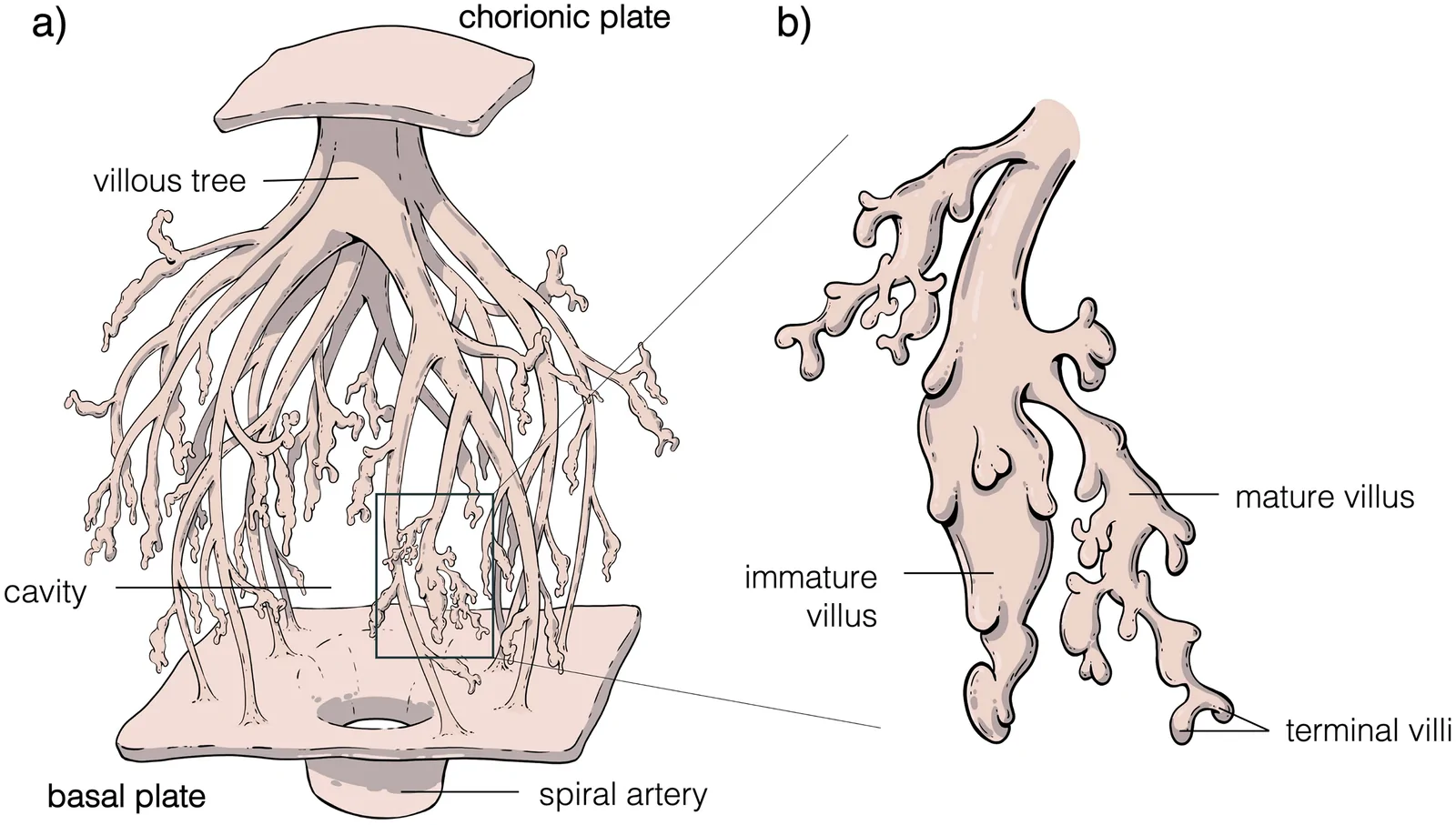
Schematic of the villous structure within a placentone, the basic functional unit of the placenta. **a)** Villous tree extending from the chorionic plate to the basal plate. **b)** Details of a dichotomous branch with immature and mature villi.

Throughout gestation, the remodeling of maternal uterine vasculature and proper development of placental villi are essential for a healthy pregnancy [1,2]. Placental villi are organized into hierarchical branching structures, known as villous trees, which mainly consist of stem villi, intermediate villi, and terminal villi [2,3]. These villous trees are the primary structures of the placenta, facilitating nutrient and gas exchange between the mother and fetus. The terminal villi, in particular, serve as the main feto-maternal exchange sites as they contain a dense fetal capillary network [2,4]. Fig 1 schematically illustrates the villous structure within a placentone, the basic functional unit of the placenta.

Humans have hemochorial placentas, where maternal blood is in direct contact with the developing fetal tissues. After the disintegration of the trophoblast plug toward the end of the first trimester, maternal blood enters the placentone through the spiral arteries (SAs), bringing the placental villi into direct contact with maternal circulation. In a healthy pregnancy, the SAs undergo extensive remodeling to slow the flow of maternal blood. This deceleration is necessary to create a suitable intervillous space (IVS) hemodynamic environment that supports efficient feto-maternal exchange and placental development [2,5]. Vascular casting [6] and *in vivo* imaging studies using magnetic resonance imaging (MRI) [7] show that there exists a free-of-villi cavity proximal to the SA opening (Fig 1). This cavity acts as a buffer zone, where blood flow decelerates further before entering the villi-containing region. However, the blood flow velocities near this cavity, which marks the interface between the free-of-villi and the dense-villi regions, remain higher than those deeper in the placentone. Notably, histological studies report terminal villi deficiency around the cavity even in healthy, at-term placentas [2], suggesting that reduced terminal villi development may be associated with hemodynamic conditions in this region.

Incomplete remodeling of SAs impairs blood flow deceleration before maternal blood enters the placentone, leading to increased IVS velocities [5,8]. High IVS velocities can damage the delicate villous structure and compromise the biochemical exchange between the mother and fetus, potentially leading to complications such as intrauterine fetal growth restriction (IFGR) and preeclampsia [1]. Histopathological studies have reported partial SA remodeling [9–11] and terminal villi deficiency [12,13] in some complicated pregnancies [14,15], such as in cases of IFGR and preeclampsia, suggesting an association between abnormal hemodynamics and impaired placental development.

The placental villi are lined with trophoblast cells, which act as the interface between the maternal and fetal blood. The development of the placenta relies on the production of specific signaling proteins and signaling molecules that drive trophoblast proliferation and differentiation, angiogenesis within the placental villi, and the remodeling of the SAs, thereby establishing an efficient maternal–fetal circulation [12,16,17]. Mechanical forces can modulate the secretion of these biochemical signals, thereby influencing cell behavior. The effect of mechanical stimulus on endothelial cell behavior has been extensively studied in vascular biology. For example, shear stress is a key stimulus for the production of nitric oxide (NO), a potent vasodilator [18,19]. Shear stress can also significantly influence the secretion of angiogenic factors such as vascular endothelial growth factor A (VEGF-A) and basic fibroblast growth factor (bFGF) [20,21] and affect receptor availability [22].

Although less extensively studied, several research studies have explored the mechanobiology of trophoblast cells. These studies indicate that shear stress can regulate trophoblast responses at multiple levels. For example, applying a shear stress of 0.1 *Pa* to syncytiotrophoblast cells increased the production of placental growth factors (PlGF) more than twice that observed under static conditions [23]. The formation of microvilli on the syncytiotrophoblast surface is essential for efficient biochemical transport between mother and fetus, as these membrane protrusions increase the available surface area for molecular exchange [24,25]. Exposure of trophoblast cells to a shear stress in the 0.0001−0.01Pa range has been shown to promote the formation and elongation of microvilli compared to the static condition. The effect of shear stress on trophoblast metabolic programming has also been examined experimentally, showing a shift in trophoblast metabolism from glycolysis toward amino-acid utilization with shear stress of 0.095 *Pa* [26].

While shear stress plays an essential role in regulating cellular processes and tissue development, levels exceeding physiological thresholds can be detrimental to cell integrity and tissue function. Physiological shear stress ranges vary by cell type. For example, under physiological conditions, shear stresses generated by normal blood flow in the venous and arterial systems typically range from 0.1 to 9.5 *Pa* [27]. However, in cases of severe arterial stenosis, shear stress may exceed 100 *Pa* [28]. The impact of such high shear stress on endothelial cells has been investigated using microfluidic systems, where flow conditions can be precisely controlled [28,29], demonstrating that elevated shear stress (16.67-100 *Pa*) can lead to rapid endothelial cell detachment, resulting in vascular injury [28]. Beyond endothelial cells, other cell types also exhibit shear stress sensitivity; for instance, experimental studies involving rat alveolar cells have shown that exposure to a shear stress of 3 *Pa* can compromise cytoskeletal integrity [30,31]. Recent work has begun to address this in the placenta: A combined computational-experimental study linked elevated fetal microvascular shear stress to placental pathology, demonstrating that increased shear stress reduced endothelial cell migration and may contribute to the impaired fetal angiogenesis observed in IFGR pregnancies [32].

Collectively, these studies underscore the influence of shear stress on cellular behavior and highlight differences in the definition of physiological shear stress. As the placenta is a growing organ, mechanical forces generated by maternal blood flow likely play a substantial role in shaping its development and function. However, there is no quantitative evidence on the physiological shear-stress range for the development of the placental microstructure. In particular, remodeling of the SAs can alter iIVS hemodynamics, which, in turn, changes the WSS acting on placental villi, potentially affecting their structure and development.

Only a few computational studies have quantified WSS on the placental villi [33,34]. However, these image-based simulation studies were limited to localized anatomical reconstructions, such as a segment of a villous branch [33] or the IVS region proximal to the SA opening [34]. Despite their high anatomical detail, these models have not always incorporated multiscale coupling to global IVS hemodynamics, potentially limiting the physiological interpretation of the predicted flow and WSS. In addition, previous studies have considered only a single placental sample, preventing assessment of microstructural variability, and focused exclusively on term placentas, precluding investigation of earlier stages of placental development. To our knowledge, no previous computational study has investigated WSS on placental villi during earlier stages of placental development, when mechanical forces may actively influence villous growth and remodeling. Consequently, the mechanistic relationship between SA remodeling and villous WSS during placental development remains poorly understood.

More broadly, multiscale computational modeling has emerged as an important approach in computational biology and biomedical engineering for integrating biological and physical processes across spatial and temporal scales and elucidating mechanisms that link local cellular and tissue-scale environments to system-level function [35–37]. This approach is particularly relevant to the placenta, where maternal hemodynamics, placental microstructure, and tissue-scale biomechanics are intrinsically coupled, collectively regulating maternal-fetal transport and oxygen exchange [38,39]; however, these interacting processes cannot yet be characterized simultaneously *in vivo* or in experimental platforms.

The concurrent occurrence of incomplete SA remodeling and terminal villi deficiency in many complicated pregnancies suggests a possible causal link. This hypothesis is further supported by the developmental timeline: SA remodeling is largely completed by the end of the second trimester [40–43], whereas terminal villi start to form at the beginning of the third trimester [2,4]. In this paper, we establish, for the first time, a connection between SA remodeling and placental villi development. We present a novel, computationally efficient framework for estimating WSS on placental villi across healthy and pathological conditions while accounting for microstructural variability. Our computational models represent the late stage of the second trimester, and we estimate WSS on placental intermediate villi from which terminal villi arise. We further analyze WSS in regions typically devoid of terminal villi in healthy pregnancies — around the free-of-villi cavity [2] — to determine a WSS range that may hinder terminal villi development. Finally, we investigate how partial SA remodeling can lead to the formation of an echogenic cystic lesion (ECL), a type of placental lake (villous-free area within the IVS) forming around the cavity, which is observed in some complicated pregnancies [44]. Under healthy conditions, smooth muscle cells are lost from the SAs as they remodel, and flow pulsatility diminishes; however, incomplete SA remodeling may lead to persistent flow pulsatility. In this study, the maternal flow is modeled under steady-flow conditions representative of the systolic phase, providing a framework for comparing the effects of SA remodeling and blood rheology on peak-flow hemodynamics, particularly maximum WSS.

The remainder of this paper is organized as follows. First, we introduce the computational framework for estimating WSS on placental villi and outline the details of the numerical simulations. Then, we present the simulation results and identify WSS thresholds relevant to placental development. Finally, we discuss the implications of these findings for understanding villous morphogenesis and placental pathophysiology.

## Materials and methods

Our computational framework employs a two-scale modeling strategy that integrates a macroscale (placentone) and a microscale (placental intermediate villi) model. The macroscale, or placentone, model employs a porous medium approach to represent the villi-containing region, as modeling the full-scale villous structure of the placentone would make the computational simulation intractable. In this approach, the influence of villous structures on blood flow is incorporated through porous-medium properties, including porosity and permeability. Here, porosity describes the fraction of the villi-containing region available for blood flow, whereas permeability characterizes the ease with which blood can move through the IVS. The main limitation of using a porous medium approach is that the macroscale model cannot provide accurate WSS estimations on the placental villi. However, from this model, we can obtain the average IVS velocities. To estimate WSS on placental villi, we created microscale models informed by statistics of the placental villi microstructure from histology and 3D synchrotron micro-CT reports. Histological and micro-CT information includes villi dimensions and IVS pore diameter distribution [2,45]. The microscale model can be positioned at any location within the villi-containing region of the placentone, ranging from areas adjacent to the cavity to deeper regions. The inlet boundary condition is defined based on the IVS velocities obtained from the macroscale model at the corresponding location. This multiscale computational framework enables efficient estimation of WSS on placental villi while accounting for microstructural variability and model uncertainty.

Details of the macroscale and microscale models are provided in the following sections.

## Placentone model (macroscale model)

### Model construction

In our previous work [8], we established an at-term physiological model for the placentone. The model consisted of a cylindrical lobule composed of a porous medium domain representing the villi-containing region and a fluid domain representing the central free-of-villi cavity, a central SA, and two decidual veins located in the lobule periphery. As the placenta is a growing organ, we adjusted our previous at-term placentone model to represent the end of the second trimester (25 weeks of gestation). The geometric specifications of the model at 25 weeks of gestation are as follows.

#### Lobule:

The lobule height was set to 18 mm based on second-trimester placental thickness measurements found in the literature [2]. Since lobule diameter measurements at 25 weeks of gestation were not directly available in the literature, we scaled the at-term lobule diameter (20 mm) used in our previous study [8] by a factor of 0.625, corresponding to the ratio of placental diameter at 25 weeks of gestation to that at term (125 mm/200 mm) [2].

#### SA:

As the remodeling of the SA is largely completed by the end of the second trimester, the same at-term SA geometry from our previous study [8] was used here to represent the healthy placentone with a fully remodeled SA. To model pathological conditions, we adapted the SA geometry to represent 0%, 25%, 50%, and 75% remodeling.

#### Cavity:

The cavity diameter was set to 3 mm to prevent flow recirculation in the healthy placentone [8], as flow recirculation can reduce the placentone efficiency [34,46]. Due to the lack of solid data on the changes in cavity size throughout gestation, we scaled the at-term cavity length using the same factor derived from the change in lobule height. This approach yielded a cavity length of 4.5 mm. This cavity length generated a jet length of 4.0 mm, consistent with physiological entry jet lengths reported at the end of the second trimester [47], as discussed in *Validation of the macroscale model* *section*.

#### Decidual veins:

Similar to the SA, we assumed that the veins were also fully developed and, therefore, we used a diameter of 1.2 mm as described in our previous work [8].

To the best of our knowledge, there is no solid data on IVS porosity at the end of the second trimester. Therefore, we adjusted the histology-based measurements of at-term porosity from the literature [48] to reflect conditions at the end of the second trimester, accounting for the change in placental volume from 25 weeks of gestation to at-term, as well as the absence of terminal villi at 25 weeks of gestation. The volume of the placenta can be estimated as ePV =−64.68+12.31×GA [49], where ePV is the placental volume in cm^3^, and GA is the gestational age in weeks. Based on this formula, the placental volume at 40 weeks of gestation (at-term) is 427.7 cm^3^. At-term porosity has been estimated to be 0.57 [48], which results in a 183.9 cm^3^ volume occupied by villi. Deducting 40% of this villi volume that represents the volume occupied by terminal villi at term [2], we obtained a porosity value of 0.55 at the end of the second trimester (25 weeks of gestation). In the absence of permeability measurements for the end of the second trimester, and given that the calculated porosity is very similar to that at term, we assumed at-term permeability (0.00162 mm^2^ [8,50],) for our simulations. This choice is supported by the Kozeny–Carman relation, an approximate, simple model for permeability in porous media, where permeability depends primarily on porosity.

**Fig 2:**
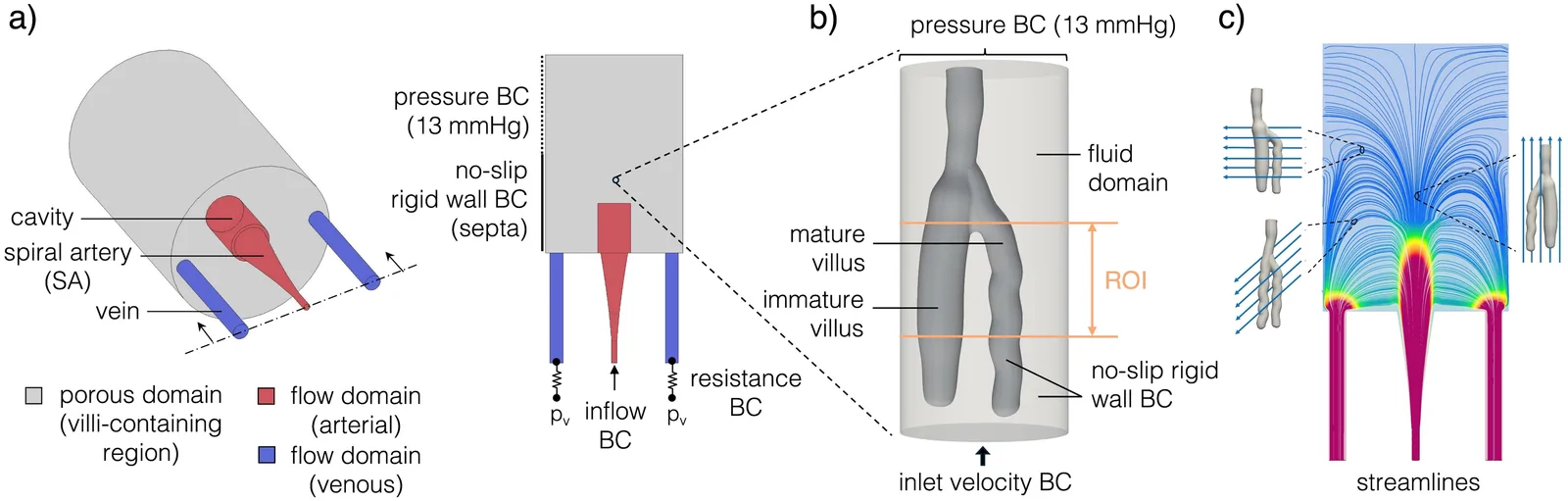
Macroscale and microscale models. **a)** Placentone (macroscale) model at the end of the second trimester and its 2D middle cross-section. **b)** An example of the microscale model. **c)** Schematic illustration of the coupling between the macroscale and microscale models.

Fig 2a shows the placentone model at the end of the second trimester and its 2D middle cross-section.

### Boundary conditions and mesh

We applied the same placentone model boundary conditions as in our previous work [8]. Briefly, a prescribed parabolic flow rate at the SA inlet, a resistance boundary condition at the venous outlets, and a pressure boundary condition at the interface with contiguous placentones. The inlet flow rate was set to a steady value of 149.5 mm^3^/*s* (*Re* = 115). A steady flow assumption is reasonable for fully remodeled SAs, as the loss of smooth muscle cells during SA remodeling results in diminished pulsatility. However, in partially remodeled SA cases, the flow pulsatility may not be negligible due to the retention of SA smooth muscle cells, which are lost during the SA remodeling process [2]. Here, we simulated the systolic phase, as it will lead to maximum WSS, and our focus was on examining the effect of high WSS on placental villi. We employed similar mesh parameters as in our previous work, obtained from a mesh independence study [8], a maximum element edge size of 0.05 mm in the SA and cavity, 0.1 mm in the veins, and 0.3 mm in the rest of the lobule [8].

### Governing equations and numerical solver

The governing equations in the fluid domain (SA, cavity, and veins) are the incompressible Continuity Eq 1 and Navier-Stokes Eq 2 equations, and in the porous domain (placentone lobule except the cavity) are the Continuity Eq 1 and Darcy-Brinkman Eq 3 equations.


$$\begin{array}{c} \nabla \cdot 𝐮=0, \end{array}$$ (1)

$$\begin{array}{c} \rho \left(\frac{\partial 𝐮}{\partial t}+𝐮\cdot \nabla 𝐮\right)=-\nabla p+\nabla \cdot \left[\mu \left(\nabla 𝐮+\nabla {𝐮}^{⊤}\right)\right], \end{array}$$ (2)

$$\begin{array}{c} \frac{\rho }{\Phi }\left(\frac{\partial 𝐮}{\partial t}+\frac{1}{\Phi }𝐮\cdot \nabla 𝐮\right)=-\nabla p+\nabla \cdot \left[\mu \left(\frac{\nabla 𝐮+\nabla {𝐮}^{⊤}}{\Phi }\right)\right]-\frac{\mu }{k}𝐮, \end{array}$$ (3)

where, 𝐮 is the fluid velocity vector, *p* is the fluid pressure, ρ and μ are the fluid density and dynamic viscosity, and Φ and *k* are the porous medium porosity and permeability, respectively. Blood flow was assumed to be Newtonian with viscosity μ=0.0035gr/mms and density $\rho =0.00106\;\text{gr}/{\text{mm}}^{3}$ [8,51]. Numerical simulations were performed using SimVascular open-source finite-element-based multiphysics solver [52,53], along with our custom implementation of the porous medium model [8]. The simulation time step was 0.001 s.

### Villi model (microscale model)

The microscale model consists of a dichotomous branch of intermediate villi within an elliptical cylinder that serves as the fluid domain surrounding the villi. Fig 2b shows an example of the microscale model consisting of a mature and immature intermediate villus. We assume that the microscale model represents a streamtube [54], defined by the local streamlines at different locations inside the placentone (Fig 2c). Under the streamtube assumption, all blood flow entering the inlet boundary in Fig 2b exits through the outlet. To satisfy the streamtube assumption, based on the IVS velocity field obtained from the macroscale simulation, the microscale model should be shorter than what is depicted in Fig 2b. However, instead of reducing the microscale model height, we defined a region of interest (ROI) within the microscale domain for analysis, where the streamtube assumption holds, ensuring WSS estimation is not influenced by boundary effects (Fig 2b). The ROI spans 30% of the total microscale model height, measured from the villi junction.

To define the location of the villi inside the ROI and the dimensions of the fluid domain, we analyzed IVS pore diameter distributions obtained from 3D synchrotron micro-CT images [45]. We fitted a gamma probability density function (shape parameter k=5.14, scale parameter θ=0.0148) to the pore diameter data (Fig 4c) and used this probability density function to randomly sample IVS pore diameters, guiding the placement of villi inside the fluid domain. All generated microscale models satisfied the average IVS pore diameter of 80±5μm within the ROI [45].

### Model construction

We built a pipeline for the automatic generation of microscale models and their corresponding finite-element mesh using Python’s OpenCascade [55] and Gmsh [56] modules. The geometric specifications of the microscale model are as follows:

#### Intermediate villi:

In the second trimester of pregnancy, both types of intermediate villi (mature and immature) are observed [2]. To evaluate how the intermediate villous type affects WSS, we constructed dichotomous branches consisting of two mature, two immature villi, and a mature and an immature villus. The diameter of the mature villus was set to 100μm [2], and its shape was modeled with undulations to reflect the characteristic bends of its longitudinal axis [2]. The diameter of the immature villus was set to 150μm without any undulations [2]. These geometrical characteristics are based on histological and descriptive reports in the literature [2]. As shown in Fig 2b, we generated a dichotomous branch originating at the distal cap of the elliptical cylinder (outlet) and extending through the fluid domain for 1.11 mm and 1.08 mm for mature and immature intermediate villi, respectively, consistent with villi geometry descriptions [2].

#### Fluid domain:

**Fig 3:**
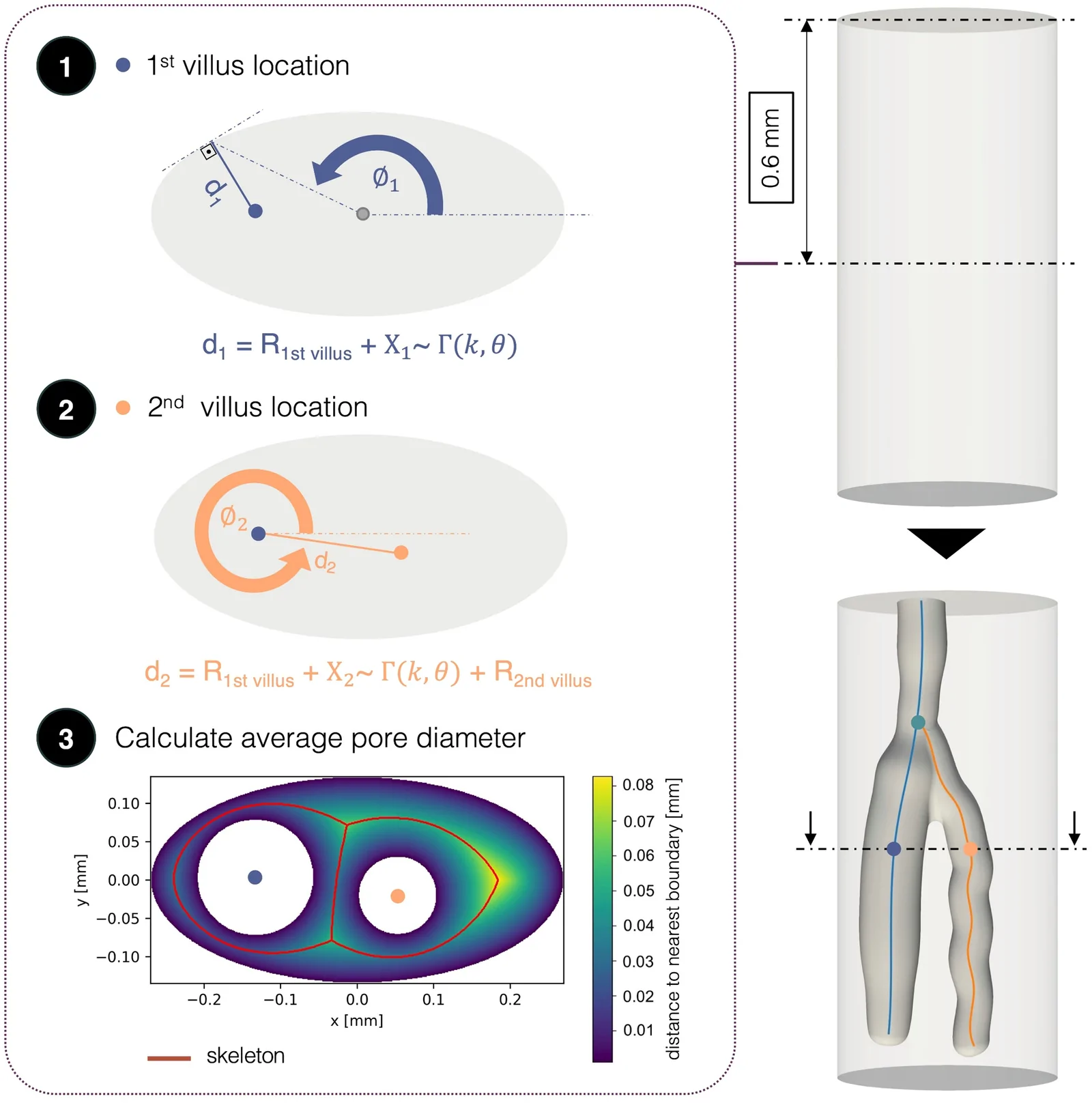
Overview of the workflow for villi placement within the fluid domain, including the distance map and the IVS pore geometry skeleton. Villi location distances d_1_ and d_2_ are defined based on the gamma probability density function, Γ(k,~θ).

The fluid domain was defined as an elliptical cylinder surrounding the villi. To prevent the inflow from becoming fully developed before reaching the villi structure, which will not be physiologically accurate, the inlet surface of the fluid domain was placed 0.03 mm upstream of the villi (Fig 2b). This entrance length was determined from computational experiments. The aspect ratio of the elliptical cross-section was set to 2 by default, to accommodate two villi forming a dichotomous branch. For each villi type group, after placement of the two villi using the pore diameter probability density function described earlier, the elliptical short axis was adjusted to ensure the average pore diameter of 80±5μm was satisfied. Consequently, all samples generated for a given villi type group shared the same elliptical cross-section. The procedure for positioning the two villi within the fluid domain was as follows: First, we selected a cross-section within the ROI and away from the junction (0.6 mm from the outlet surface). The first villus was randomly placed within this plane according to the pore diameter probability density function. The second villus was then placed relative to the first villus by again sampling from the same distribution and evaluating the resulting pore size. Each time, an angle was randomly selected to set the placement direction. To calculate the average IVS pore diameter at the specified cross-section, we developed a 3-step algorithm: (1) A distance map at the cross-section was generated, giving the in-plane distances from each point to its nearest solid boundary (either the external periphery or the interior villus boundaries). (2) The skeleton of the IVS pore geometry was extracted to obtain the centerlines within the pore domain. (3) Distances sampled along the skeleton provided local pore radii and were converted to local pore diameters. The average of these pore diameters was taken as the average IVS pore diameter of the model. Fig 3 shows the distance map and its corresponding skeleton (red) for a microscale model. Finally, additional path points for each villus were generated from the randomly defined locations at the specified cross-section. The relative distances of the junction and the undulations were defined based on descriptions of villi geometry in the literature [2]. The villi generation workflow is illustrated in Fig 3.

### Boundary conditions and mesh

The boundary conditions applied to the microscale model are illustrated in Fig 2b. The inlet velocity boundary condition was set to match the IVS velocity obtained from the macroscale model simulation at the exact location where the microscale model was evaluated. As explained earlier, the macroscale model provides the average IVS velocity in the villi-containing region, whereas the microscale model explicitly includes the villous geometry and resolves local velocity variations around the villi. The microscale model inlet boundary condition, therefore, serves as the interface between the homogeneous IVS macroscale representation and the fully resolved microscale IVS geometry. We imposed a flat velocity profile to preserve the predicted average velocity by the macroscale model without introducing additional assumptions regarding the inlet velocity profile. To evaluate the sensitivity of the WSS estimation to the choice of inlet velocity profile, we compared simulation results obtained with both a flat and a parabolic velocity profile, while preserving the same average inlet velocity. For an IVS velocity of 3 mm/*s*, the parabolic profile changed the mean WSS in the ROI by less than 0.05% compared with the flat profile, and this difference was not statistically significant (*p* = 0.993). Once the blood enters the microscale domain, the flow is redistributed according to the villous geometry. Consequently, the local velocity and WSS patterns are governed primarily by flow pathways created by the villi structure rather than by the initial inlet velocity profile shape. The outlet boundary condition was set to a physiological IVS pressure of 13 mmHg [8]. A no-slip rigid wall boundary condition was applied on the villi surface. Since the model includes only a dichotomous branch of villi, we approximated the influence of neighboring villi, which are not explicitly present in the model, by imposing a no-slip rigid-wall boundary condition along the periphery of the fluid domain.

To mesh the model, we used tetrahedral elements with local mesh refinement applied around the villi. The maximum element edge size in the fluid domain was set to 0.015 mm, refined near the villi wall to 0.00275 mm with a 0.02 mm transition layer thickness. These mesh parameters were defined based on a mesh convergence study. We defined convergence as a difference of less than 1% in the area-weighted mean WSS magnitude within the ROI.

### Governing equations and numerical solver

Continuity Eq 1 and Navier-Stokes Eq 2 equations govern blood flow around the villi in the microscale models. To solve these equations, we used SimVascular open-source finite-element-based multiphysics solver [52,53]. We simulated steady flow and modeled blood as a Newtonian and a non-Newtonian fluid to examine how blood’s shear-thinning behavior influences the estimated WSS across different IVS velocities. To model blood as a non-Newtonian fluid, we used the Carreau-Yasuda model Eq 4.


$$\begin{array}{c} \mu (\dot{\gamma })=\mu_{\infty }+(\mu_{0}-\mu_{\infty }){\left[1+(\lambda \dot{\gamma }{)}^{a}\right]}^{\frac{n-1}{a}}, \end{array}$$ (4)

where $\dot{\gamma }$ is the shear rate, n is the power-law index (*n* = 0.2128), λ is the characteristic time (λ=8.2s), *a* is the Yasuda parameter (*a* = 0.64), $\mu_{0}$ is the zero–shear viscosity ($\mu_{0}=0.1600\text{ Pa}\;\cdot \;\text{s}$), and $\mu_{\infty }$ is the infinite–shear viscosity ($\mu_{\infty }=0.0035\text{ Pa}\;\cdot \;\text{s}$) [57]. The simulation time step was 0.0001 s.

## Validation

### Validation of the macroscale model

We validated the healthy placentone simulation results using Doppler ultrasound data from a previous clinical study on a cohort of 52 women with normal pregnancy outcomes [47]. The study includes data from 11−13 to 33−34 weeks of gestation. We compared the model’s entry jet length and maximum velocity at the SA opening with the corresponding experimental measurements at the end of the second trimester. The length of the jet was defined as the length extending from the SA opening along the centerline until the velocity magnitude was less than 14 mm/s [8,47]. The healthy placentone model with a cavity length of 4.5 mm generated an entry jet length of 4.03 mm. This is consistent with the 4.0 mm entry jet length predicted by the regression model proposed by the authors, fitted to the Doppler ultrasound measurements at the end of the second trimester. In addition, 4.0 mm was reported as the most frequently observed entry jet length at this gestational age in the cohort. The model also produced a physiological maximum velocity at the SA opening (140 mm/*s*) consistent with peak systolic velocities measured by Doppler ultrasound. Finally, the model’s average IVS pressure of 13 mmHg is within the physiological range of 10−15mmHg [58,59].

### Validation of the microscale model

**Fig 4:**
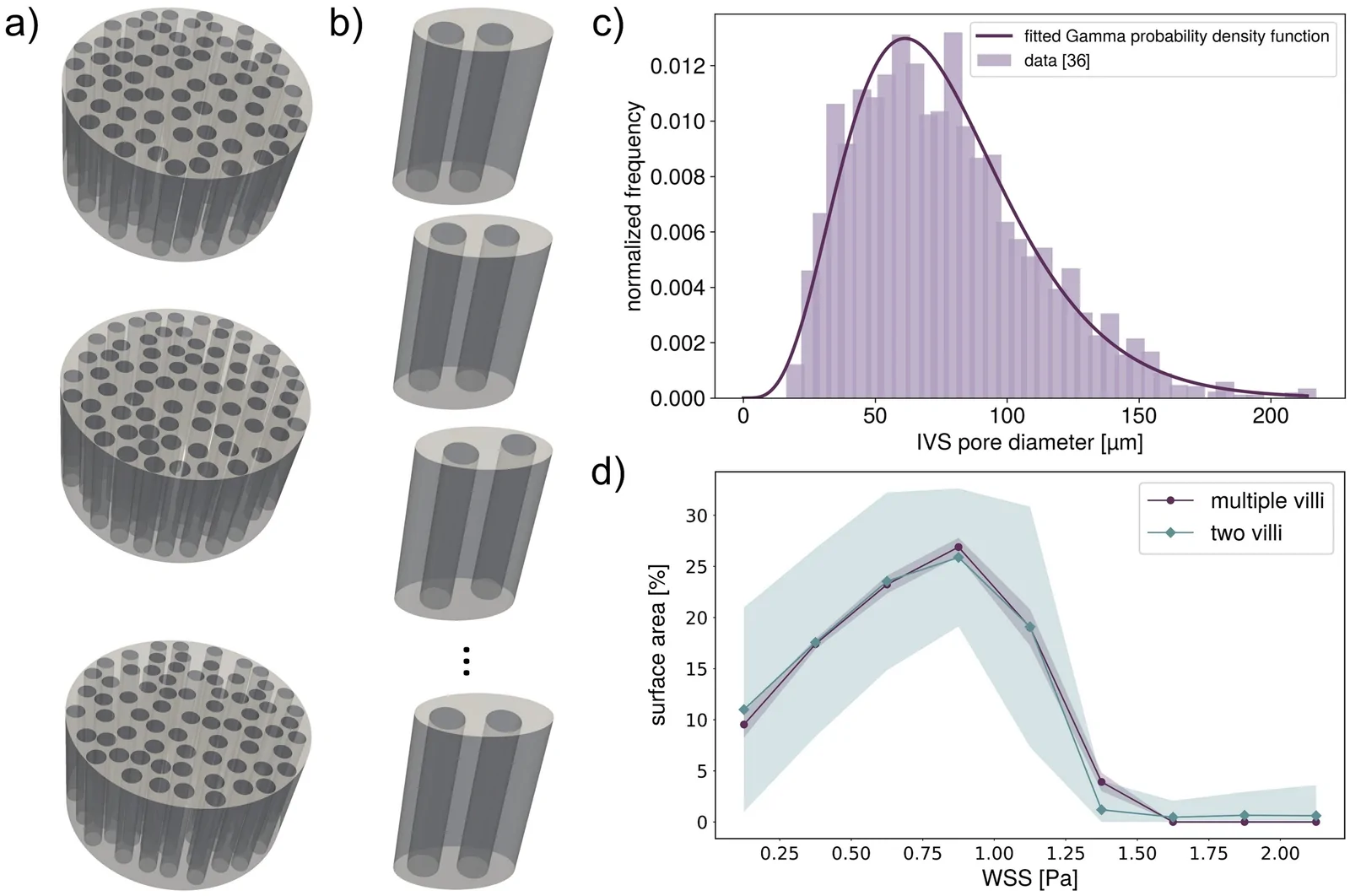
Summary of microscale model validation. **a)** Multiple-villi models. **b)** Two-villi models. **c)** IVS pore diameter distribution and gamma probability density function (shape parameter k=5.14, scale parameter θ=0.0148). **d)** WSS area distributions estimated from the two-villi models (*n* = 25) versus the multiple-villi models (*n* = 3) for an IVS velocity of 3 mm/*s*. The shaded band indicates the standard deviation among the corresponding model samples.

We conducted a series of computational experiments to assess whether our proposed multiscale modeling approach, consisting of modeling a simple dichotomous branch of intermediate villi, was sufficient to estimate WSS on the placental microstructure. To test this, we simulated blood flow in a larger portion of the placental microstructure and compared its resulting WSS area distribution with that obtained using the proposed intermediate villi model. For simplicity, in this validation step, villi were approximated as straight cylinders. These cylinders were randomly positioned within the fluid domain according to the previously defined probability density function (Fig 4c), ensuring the average IVS pore diameter was maintained. Fig 4a and 4b show the two model configurations used for validation: models capturing a larger portion of the placental microstructure (multiple-villi model) and models containing two intermediate villi (two-villi model).

To account for model generation variability, we considered the aggregate effect of multiple samples in each of the two groups, the multiple-villi and two-villi models. Because greater variability was observed among the results of the two-villi models, a larger number of samples was required in this case to achieve consistency in the aggregate result compared with the multiple-villi group. Our two-villi model analysis demonstrated that aggregate WSS estimation became consistent after the 25^th^ sample, adding another sample changed the aggregate mean and modal WSS by less than 1.2% and 0.2%, respectively. Therefore, we concluded that 25 samples were sufficient for our analysis. We considered only three samples of the multiple-villi model since the mean and modal WSS predictions differed by less than 1.1%. Fig 4d shows the aggregate WSS area distribution over the villi surface inside the ROI, for the two-villi model (*n* = 25) versus the multiple-villi model (*n* = 3). As expected, the microscale models with only two villi exhibited a larger standard deviation than those with multiple villi. However, their aggregate WSS area distribution was similar (Fig 4d). The mean WSS estimated from the aggregate analysis of the multiple-villi and two-villi models differed by less than 1% (0.716 *Pa* vs. 0.723 *Pa* for an IVS velocity of 3 mm/*s*). A t-test demonstrated no statistically significant difference between estimated WSS from the two-villi and the multiple-villi model (*p* = 0.86). These results demonstrate that, with a sufficient number of randomly generated two-villi microscale models, the aggregate WSS area distribution can represent the WSS on the placental microstructure.

Unfortunately, no experimental WSS measurements on placental villi are available to directly validate the microscale model results. However, the WSS ranges predicted by the model for a healthy placentone align with the physiological shear stress ranges previously employed in experimental studies investigating trophoblast cell responses to WSS [23,25]. WSS model predictions are also consistent with previously reported WSS estimates from computational simulations (0.29−0.33Pa vs. 0.23 *Pa* for an IVS velocity of 1 mm/s) [33]. In the *Discussion* section, we further compare our predicted WSS to estimates from computational models and experimentally tested WSS ranges known to influence trophoblast cell behavior.

### Computational experiments

We conducted various computational experiments to account for possible physiological variations that can affect WSS estimations in healthy and diseased conditions (i.e., incomplete SA remodeling).

### Villi orientation

The flow may impinge on the villi surface in different directions depending on their location in the placentone lobule or their orientation in the villous tree. To account for these variabilities, in addition to parallel flow, we also tested cases where the villi were rotated 45 degrees and were perpendicular to the flow. For each orientation, 25 microscale models were randomly generated as described previously to estimate WSS.

### Villi type

In the second trimester of pregnancy, different types of intermediate villi, mature and immature villi, can be present [2]. Therefore, to examine how different villus types can influence local WSS, three configurations were considered: a dichotomous branch of a mature and an immature villus, two mature villi, and two immature villi. For each configuration, 25 microscale models were randomly generated as described previously to estimate WSS.

First, we simulated parallel flow over a dichotomous branch of a mature and an immature villi model, modeling blood as both a Newtonian and a non-Newtonian fluid. Based on these experiments, we determined which rheological model to use in subsequent simulations.

### Data analysis

For each IVS velocity corresponding to different locations in the placentone and different levels of SA remodeling, we calculated WSS in the ROI for all 25 randomly generated samples. For each mesh element on the villous surface (triangular element), the average WSS magnitude of its three nodes was considered as the element WSS. To show the WSS area distribution on the villi surface within the ROI, we provided an area-weighted WSS distribution histogram (0.25−Pa bins; percent of total villous surface area per bin), as the curve obtained by linear interpolation between histogram bin centers. We also calculated the area-weighted mean WSS, referred to as the mean WSS from here on, and the maximum observed WSS.

To evaluate statistical differences in mean WSS estimates across IVS velocities, villi orientations, and villi types, we performed two-sample t-tests. For each comparison, the data in each group consisted of the mean WSS values calculated from the individual microscale models.

As described previously, a natural terminal villi deficiency is observed around the cavity in a healthy placentone [2]. We assume that this deficiency results from elevated WSS acting on the villi around the cavity, driven by higher IVS velocities relative to those in deeper regions. Under this assumption, by calculating the WSS around the cavity in a healthy placentone, we define a WSS range that may hinder terminal villi development.

Given the delicate villi structure, the natural absence of villi proximal to the SA opening can be due to the high momentum blood flow at the SA opening as the maternal blood enters the placentone. The velocities observed within the cavity of a healthy placentone are therefore interpreted as indicative of flow conditions capable of inducing villous tissue loss. Based on this interpretation, we analyze the IVS velocity field in incomplete SA remodeling cases to identify regions prone to placental lake formation.

## Results

### Incomplete SA remodeling results in increased blood velocity inside the placentone

**Fig 5:**
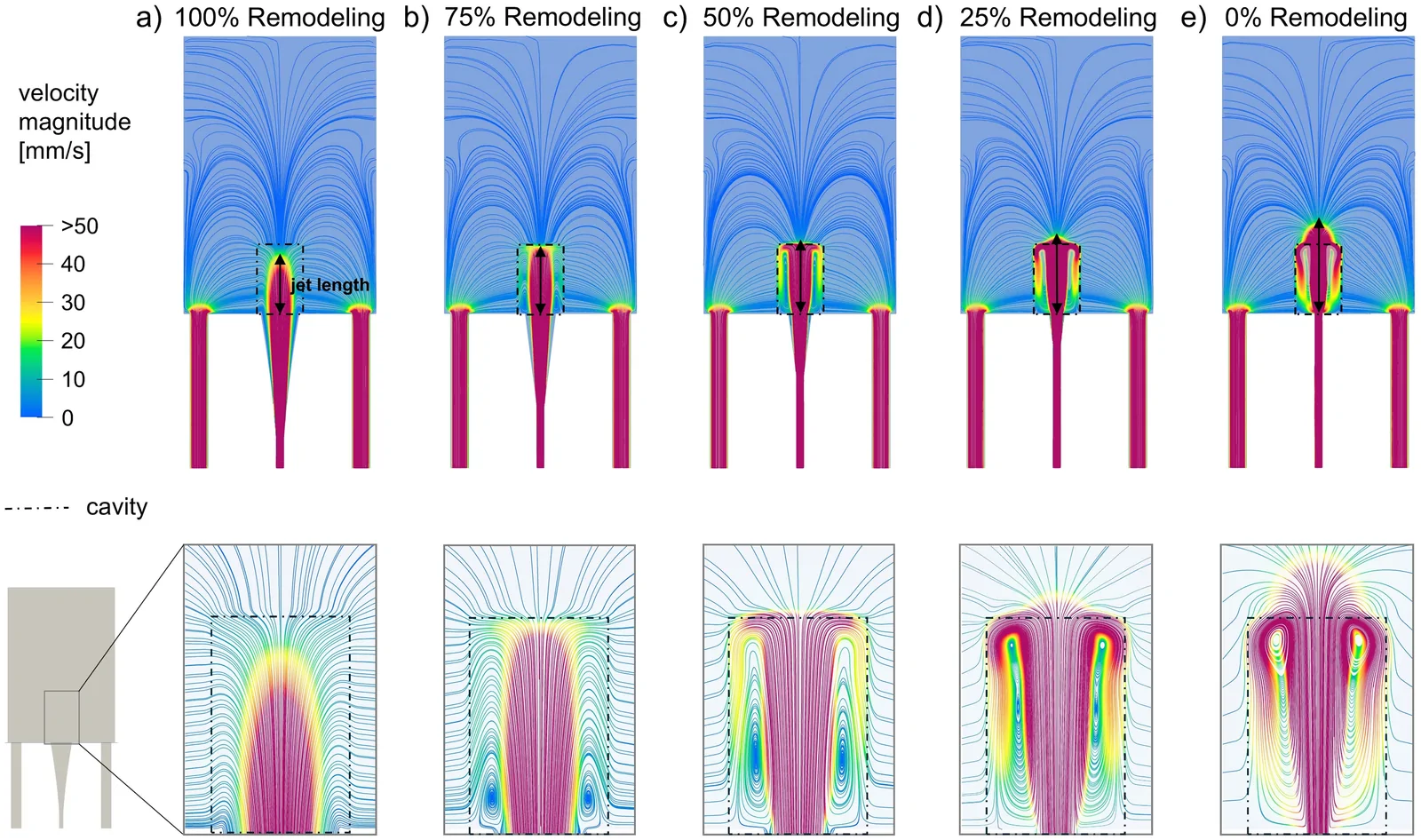
Effect of SA remodeling on blood velocity inside the placentone. Panels **a)** to **e)** show the velocity magnitude and streamlines for different degrees of SA remodeling.

The macroscale placentone simulations demonstrate that as we move away from the basal plate, deeper into the placentone, blood velocity decreases. We also observe that incomplete SA remodeling leads to higher velocities in the placentone lobule (Fig 5). For example, the maximum velocity in the placentone with a fully remodeled SA is 6 mm/*s*. However, the maximum velocities in 75%, 50%, 25%, and 0% SA remodeled cases increase to 15, 58, 172, and 589 mm/*s*, respectively. In all the different SA remodeling cases tested, the maximum velocities are observed at the top of the free-of-villi cavity (Fig 7b). In the fully remodeled SA case, the maximum velocity is observed at the corner of the cavity. As SA remodeling decreases, the location of the maximum velocity moves towards the center of the cavity (Fig 7b). In Fig 5, it can also be observed that with less SA remodeling, the maternal blood flow jet length increases and flow recirculation emerges.

### Newtonian blood flow assumption significantly underestimates WSS for low IVS velocities

**Fig 6:**
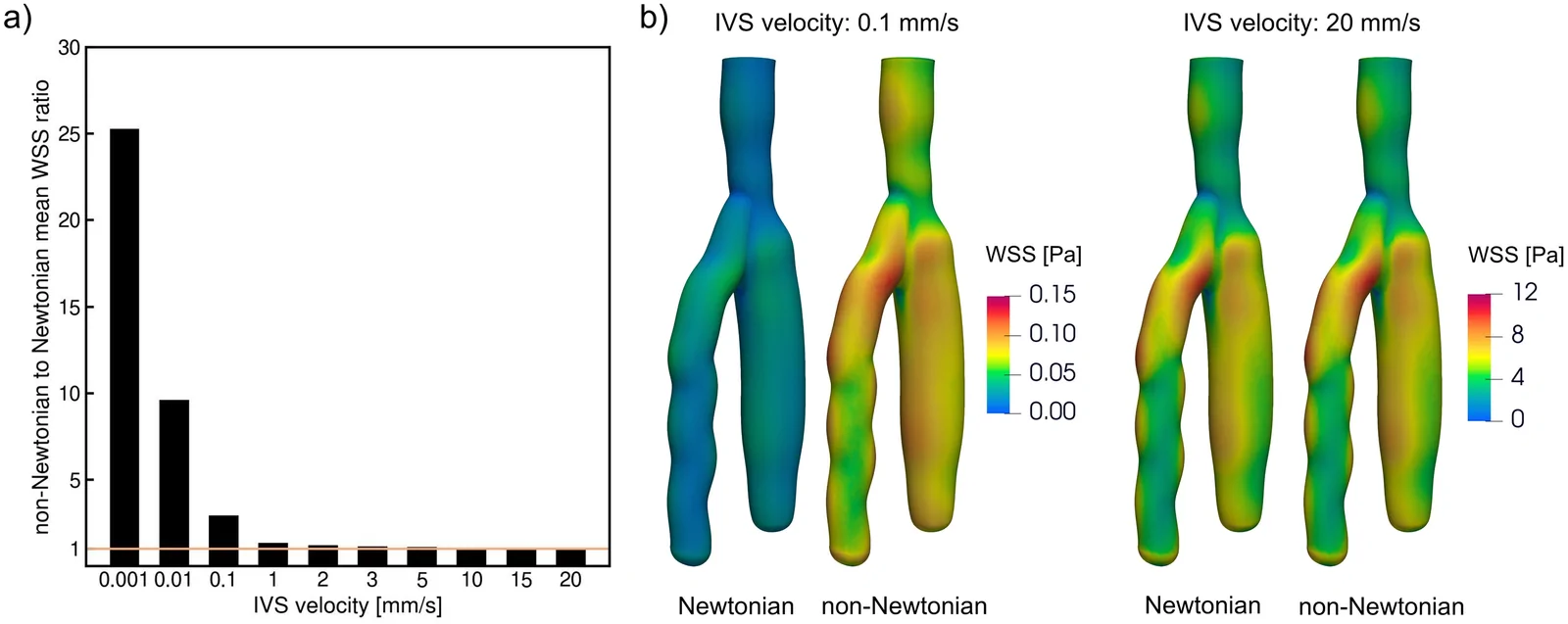
Effect of non-Newtonian blood flow model on mean WSS estimation. **a)** Ratio of non-Newtonian to Newtonian mean WSS for different IVS velocities. **b)** Three-dimensional WSS distribution on a placental villi model for two different IVS velocities (0.1 mm/*s* and 20 mm/*s*) obtained using a Newtonian and a non-Newtonian blood flow assumption.

**Fig 7:**
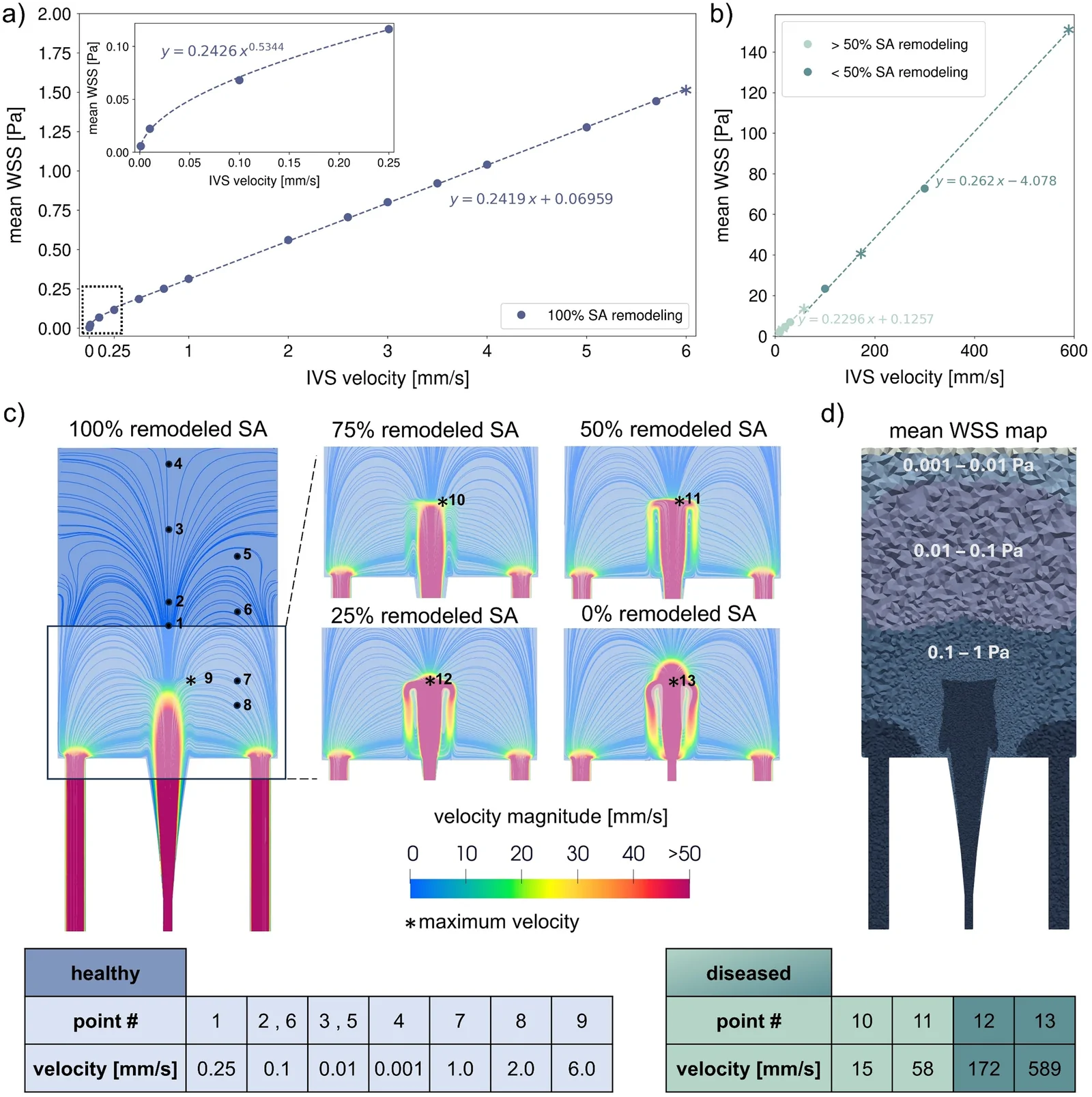
Effect of IVS velocity on placental villi mean WSS. **a)** Mean WSS versus IVS velocity in a healthy placentone. **b)** Mean WSS versus IVS velocity observed only in incomplete SA remodeling cases. **c)** Map of IVS velocity for complete and incomplete SA remodeling. **d)** Placental villi mean WSS map in a healthy placentone.

To link IVS velocity to the WSS acting on placental villi, we first examined how simulation results differed when blood was modeled as a Newtonian versus a non-Newtonian fluid. In a healthy placentone, the IVS velocity can range from 6 mm/*s* as the maximum observed velocity at the top of the free-of-villi cavity to very small velocities such as 0.001 mm/*s* deep in the placentone (Fig 7c). Fig 6 shows the ratio of the non-Newtonian to Newtonian mean WSS estimation for different IVS velocities. Large WSS differences can be observed between Newtonian and non-Newtonian models at low IVS velocities. For IVS velocities below 5 mm/*s*, the difference between the two models exceeds 10%. However, this difference becomes negligible for higher IVS velocities. We observe less than 10% difference in mean WSS estimation for IVS velocities greater than 5 mm/*s*, and less than 5% difference for IVS velocities greater than 15 mm/*s*. Therefore, in the following sections, for simulations with IVS velocities greater than 15 mm/*s*, we used a Newtonian model; for velocities less than 15 mm/*s*, we employed a non-Newtonian model.

### Placental villi are exposed to higher shear stresses in cases of incomplete SA remodeling

Fig 7a shows mean WSS for the range of IVS velocities in a healthy placentone with fully remodeled SA, and Fig 7b shows mean WSS corresponding to IVS velocities which are observed only in the pathological cases. Each data point represents the aggregate results from 25 mature and immature intermediate villi dichotomous branch model samples subjected to parallel flow. For IVS velocities less than 0.25 mm/*s*, a power-law model provides a suitable fit with a root mean squared error (RMSE) of 0.0016 (Fig 7a). For higher IVS velocities (> 0.25 mm/*s*), linear models were used to represent WSS across different IVS velocity ranges of 0.25 to 6 mm/*s*, 6–58 mm/*s* (only observed in pathological cases with > 50% remodeling), and 58–589 mm/*s* (only observed in pathological cases with < 50% remodeling). The RMSE values were calculated as the root mean squared residuals between the simulated mean WSS values and the fitted model predictions for the same IVS velocities. The proposed linear fits are:

- 0.25≤v≤6 mm/s, *y* = 0.2419*x* + 0.0696 (RMSE = 0.0064),
- 6<v≤58 mm/s, *y* = 0.2296*x* + 0.1257 (RMSE = 0.0544),
- 58<v≤589 mm/s, y=0.2620x−4.078 (RMSE = 1.1417).

Higher IVS velocities resulting from incomplete SA remodeling subject the placental villi to greater WSS (Fig 7b). The maximum observed velocity in a healthy placentone can generate a mean WSS of 1.51 *Pa*, while the observed maximum velocities in each of the 75%, 50%, 25%, and 0% SA remodeling cases can produce mean WSS of 3.63, 13.48, 40.71, and 151.00 *Pa*, respectively. Fig 7c shows the location of the maximum IVS velocity in each case.

Based on the data presented in Fig 7a, we generated a mean WSS map in a healthy placentone (Fig 7d). Our simulation results suggest that in a healthy placentone, around 11% of the villi-containing region is exposed to a mean WSS range of 0.001−0.01Pa, 46% to a 0.01−0.1Pa range, and 29% to a 0.1−1Pa range. These percentages were determined using a threshold filter that retains only elements whose node values lie within the specified WSS range, thereby excluding any element with even one node outside that range.

**Fig 8:**
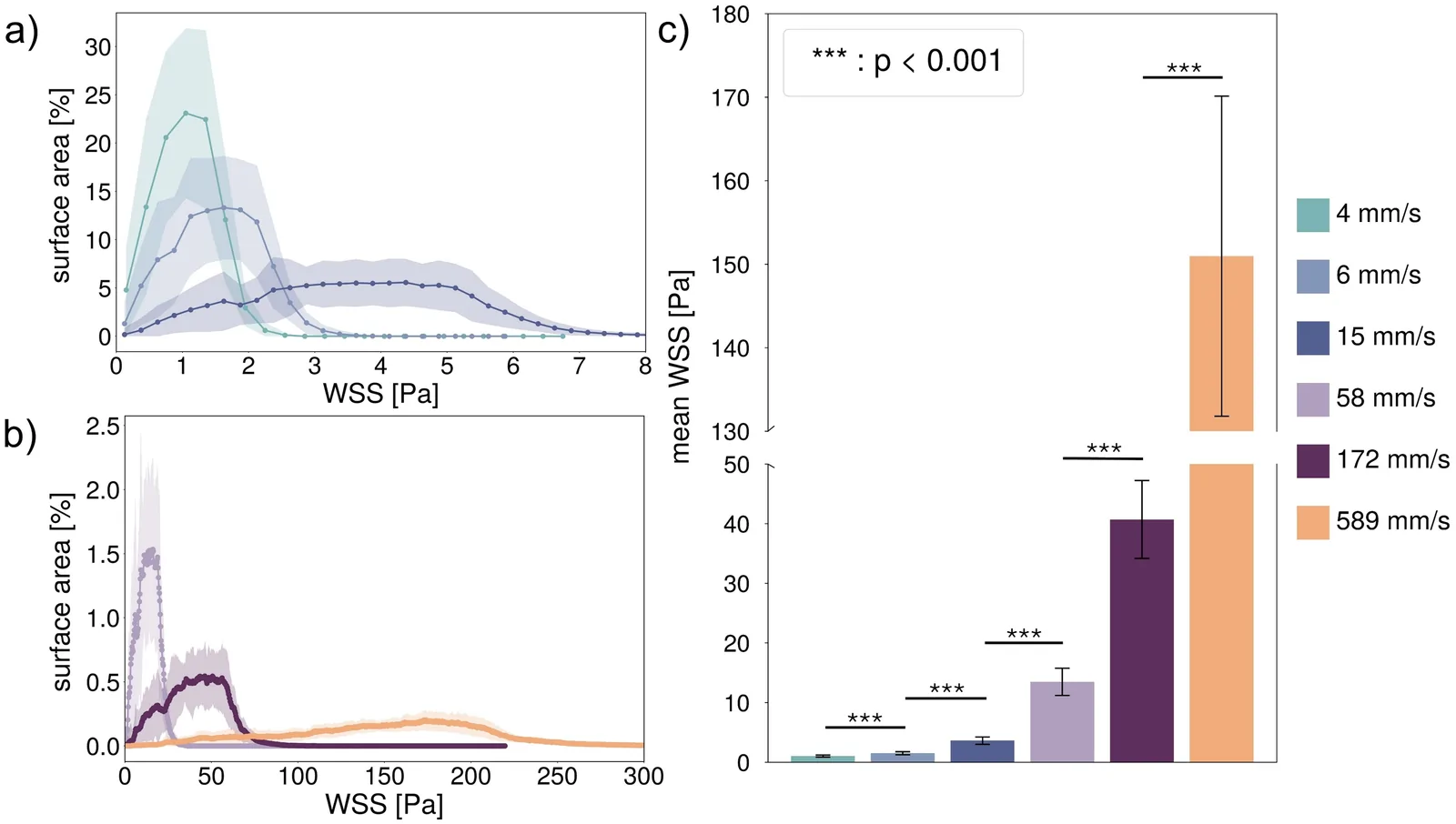
Effect of IVS velocity on villous WSS. **a)** WSS area distribution for mean IVS velocity (4 mm/*s*) around the cavity in a healthy placentone and maximum IVS velocities for fully remodeled (6 mm/*s*) SA, and 75% SA remodeling (15 mm/*s*). **b)** WSS area distribution for maximum IVS velocities observed in 50% (59 mm/*s*), 25% (172 mm/*s*), and 0% (589 mm/*s*) SA remodeling. The shaded band indicates the standard deviation among the 25 model samples. **c)** Mean WSS comparison across IVS velocities for healthy and incomplete SA remodeling cases.

Fig 8a and 8b show the WSS area distribution on the villi surface for the mean IVS velocity around the cavity in a healthy placentone (4 mm/*s*) and for the maximum IVS velocity in cases of fully remodeled SA (6 mm/*s*) and 75% (15 mm/*s*), 50% (59 mm/*s*), 25% (172 mm/*s*), and 0% (589 mm/*s*) SA remodeling. As can be seen, higher IVS velocity leads to a wider WSS area distribution. Fig 8c shows the mean WSS and standard deviation for the same IVS velocities. Mean WSS differences were statistically significant (*p* < 0.001) when comparing mean IVS velocity (4 mm/*s*) around the cavity and maximum IVS velocity (6 mm/*s*) in a healthy placentone, as well as comparing between maximum velocities for decreasing remodeling degrees (Fig 8c).

### The effect of villous orientation with respect to the flow on mean WSS is less than the effect of IVS velocity

**Fig 9:**
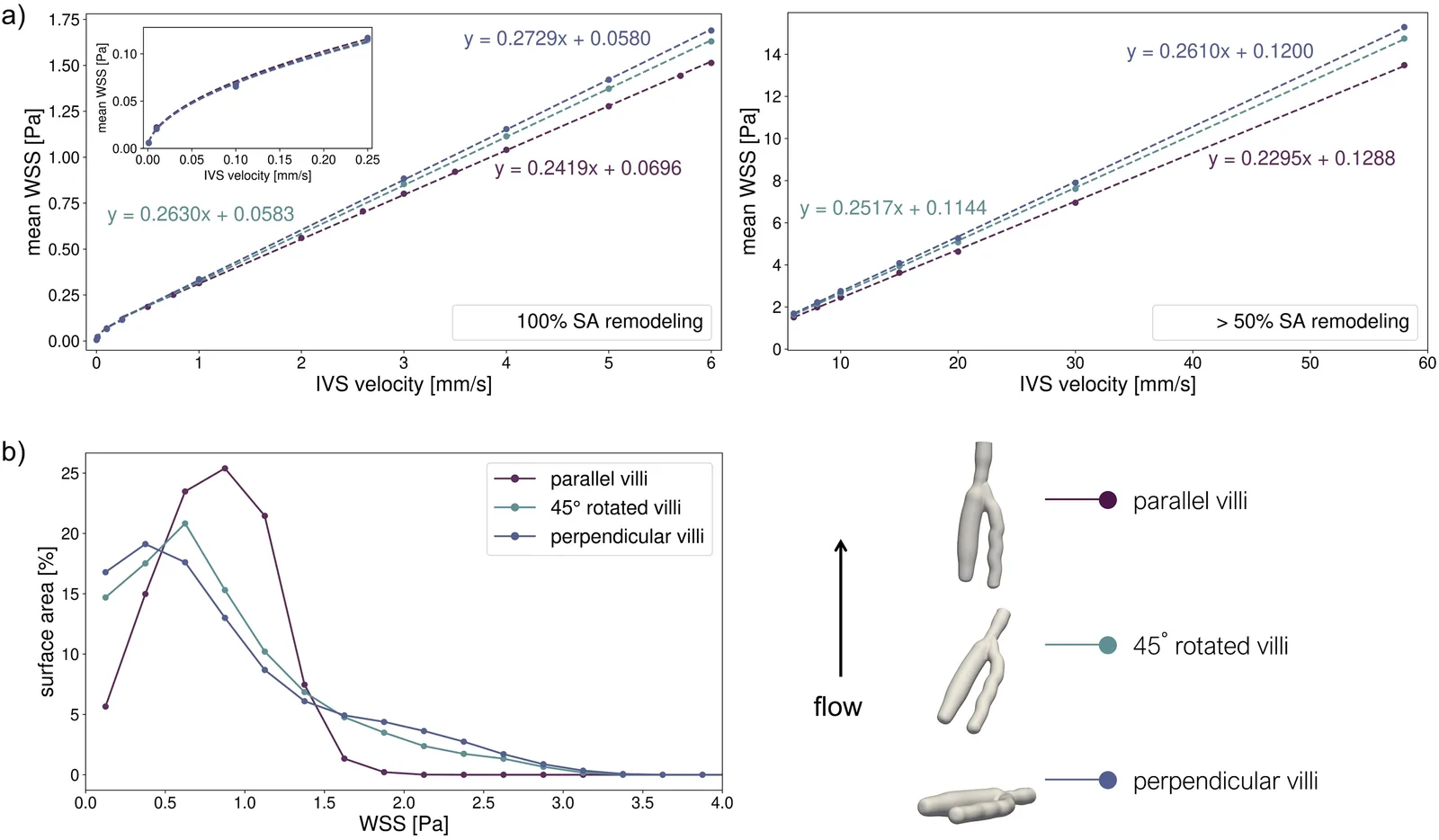
Effect of different villi orientation with respect to the flow on WSS. **a)** Mean WSS versus IVS velocity for different villi orientation angles. **b)** WSS area distribution for different orientation angles and IVS velocity of 3 mm/*s*, which represents a physiological velocity observed around the cavity in a healthy placentone.

Fig 9a shows the mean WSS versus the IVS velocity at different orientation angles between the villi branch longitudinal axis and the flow. Each data point represents the aggregate results from 25 dichotomous branch model samples corresponding to each of the three different orientation angles. While for an IVS velocity of 1 mm/*s*, we identified no significant difference between any pair of the three orientations (*p* > 0.05), for higher IVS velocities (> 3 mm/*s*), we observed statistically significant differences between the perpendicular case compared to the parallel flow case (*p* < 0.05). However, from the results presented in Fig 9a, we observe that the rate of mean WSS change between different villi orientations is approximately an order of magnitude less than the changes in mean WSS with respect to IVS velocity. Fig 9b shows the WSS area distribution for the three different orientation angles and IVS velocity of 3 mm/*s*, which represents a physiological velocity observed around the cavity in a healthy placentone.

### Villous type (mature vs. immature) does not have a significant effect on mean WSS compared to the change in IVS velocity

**Fig 10:**
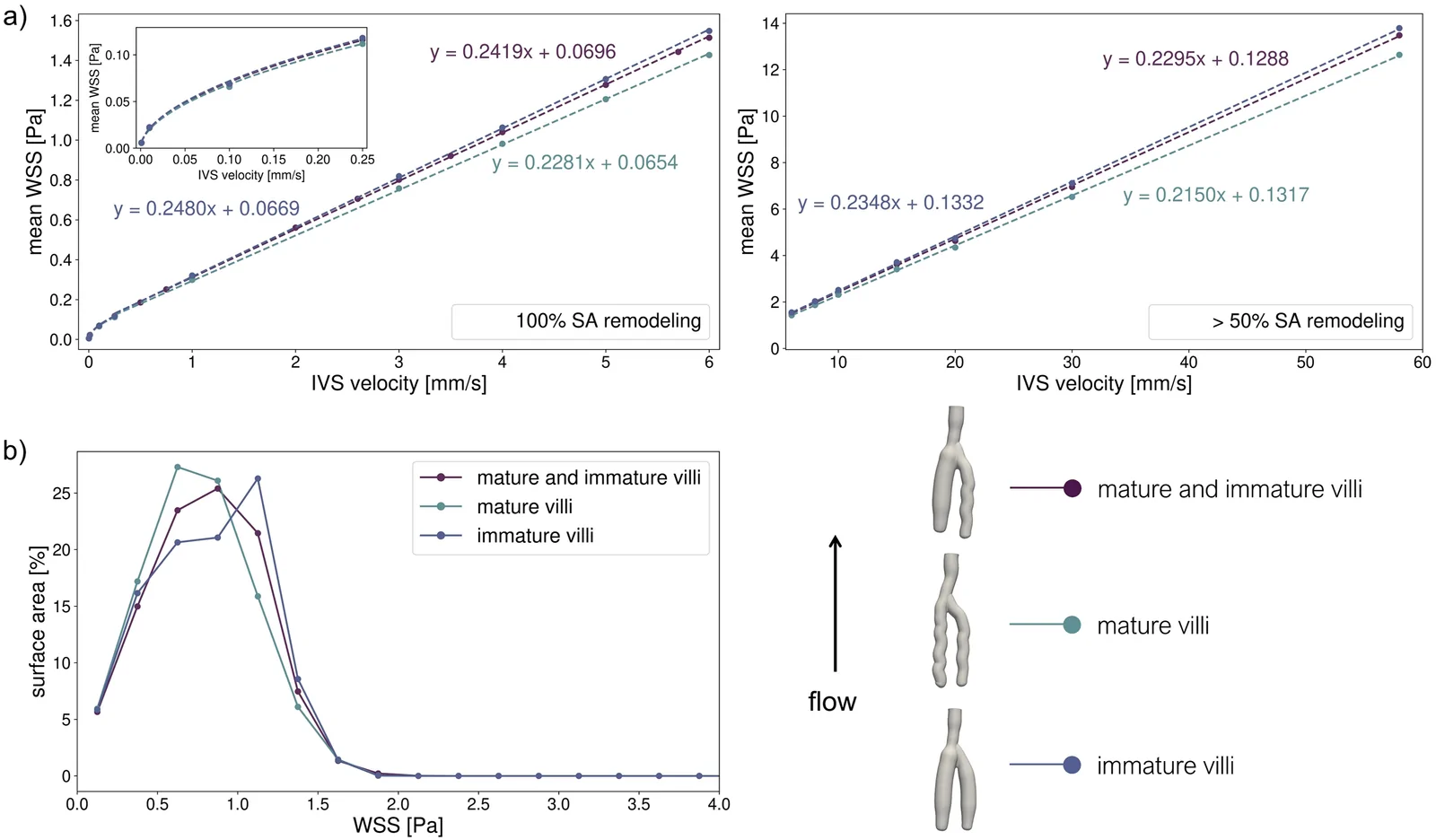
Effect of villous type on WSS. **a)** Mean WSS versus IVS velocity for different villous types. **b)** WSS area distribution for different villous types and IVS velocity of 3 mm/*s*, which represents a physiological velocity observed around the cavity in a healthy placentone.

**Fig 11:**
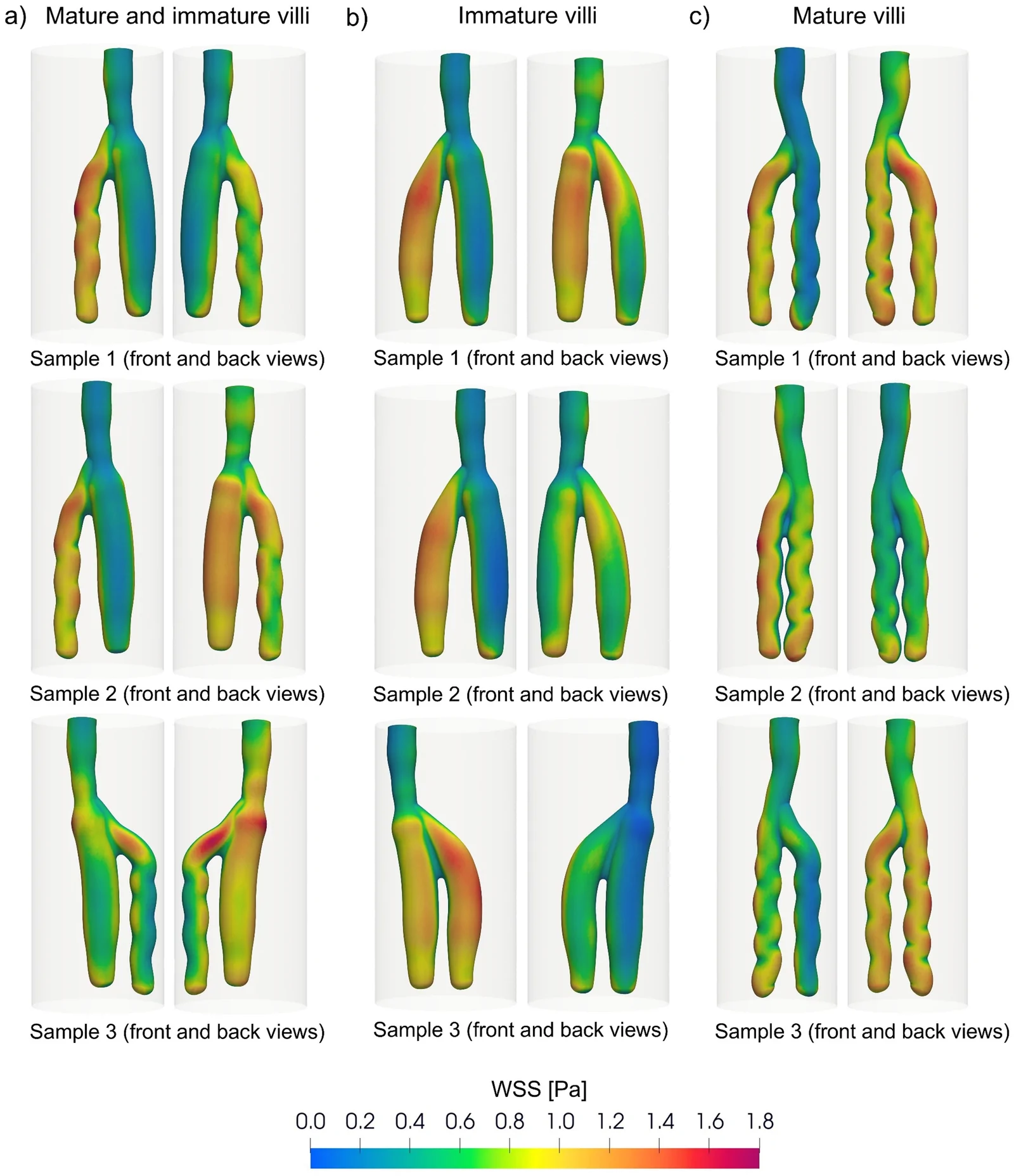
WSS contours for different villi type configurations. Three samples are shown for each configuration of **a)** mature and immature, **b)** immature, and **c)** mature intermediate villi and IVS velocity of 3 mm/*s*, which represents a physiological velocity observed around the cavity in a healthy placentone.

Fig 10a shows the mean WSS versus the IVS velocity for a dichotomous branch of two mature, two immature, and a mature and an immature villi. Each data point represents the aggregate results from 25 dichotomous branch model samples corresponding to each of the three different villous configurations tested. The rate of mean WSS change between the three different configurations is at least an order of magnitude less than the observed changes in mean WSS with respect to IVS velocity. For a given IVS velocity, we did not observe statistically significant differences in mean WSS among any pair of the three different configurations (*p* > 0.05). Fig 10b shows the WSS area distribution for the three different villous configurations and IVS velocity of 3 mm/*s*, which represents a physiological velocity observed around the cavity in a healthy placentone. Fig 11 depicts three-dimensional WSS distributions for three representative examples out of the 25 samples in each configuration, also for an IVS velocity of 3 mm/*s*.

### What WSS can hinder the formation of terminal villi?

**Fig 12:**
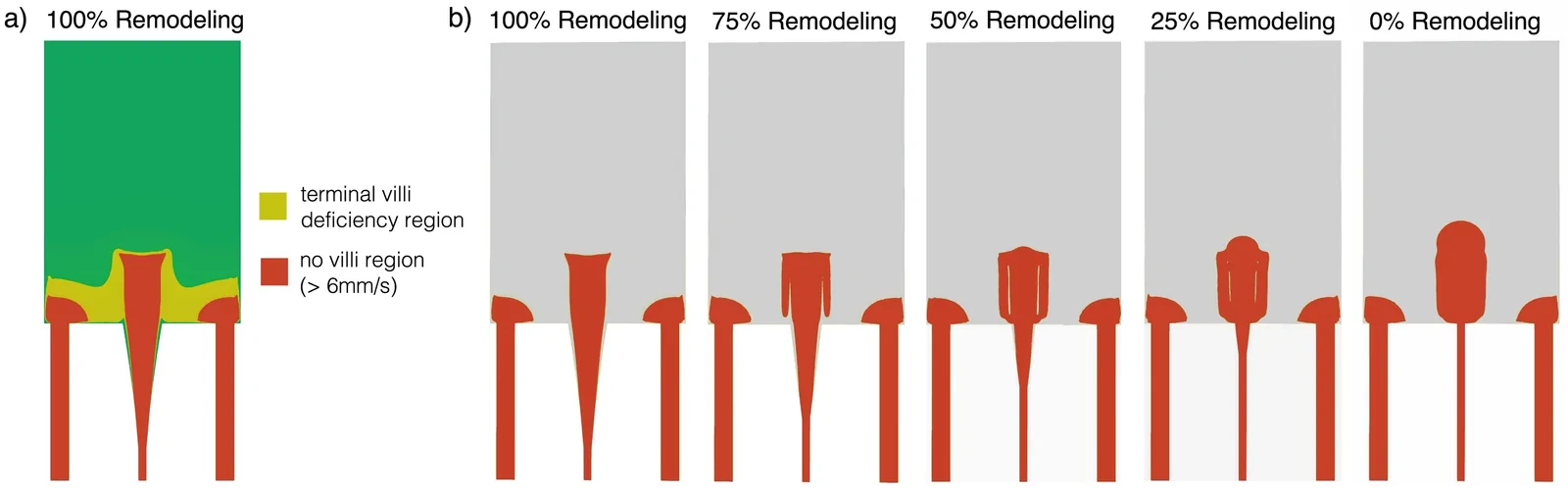
Regions of potential terminal villi deficiency and villous tissue loss. **a)** Terminal villi deficiency is observed around the cavity in the healthy placentone. **b)** ECL formation can be observed in severe, incomplete SA remodeling cases.

We analyzed blood velocity around the cavity to determine the expected mean WSS range in this region. We extracted the velocities at the surface of the cavity (mean of 4.11 mm/*s* with standard deviation of 0.77 mm/*s*) and defined a velocity range as the mean ± 2 standard deviations to include 95% of the velocities measured. Therefore, we defined the range of velocity around the cavity in a healthy placentone as 2.6 mm/*s* - 5.7 mm/*s*. These velocities can generate a mean WSS of 0.71−1.44Pa, which we propose as the range of mean WSS that can potentially hinder the formation of terminal villi. In Fig 12a, regions in yellow indicate areas within this mean WSS range in a healthy placentone, and therefore areas of potential terminal villi deficiency.

In Fig 12b, we illustrate high IVS velocity regions in incomplete SA remodeling cases. Regions in orange indicate areas with IVS velocities exceeding 6 mm/*s*. In the healthy case, these velocities are observed only within the cavity and therefore can be associated with free-of-villi regions. It can be seen that incomplete SA remodeling qualitatively correlates with larger orange regions.

## Discussion

Mechanical stimuli play a crucial role in modulating cell behavior, as well as tissue and organ development. This principle also applies to the placenta; however, fluid shear stresses acting on developing placental villi, and therefore on the syncytiotrophoblast cells, have not been previously characterized. In this study, we present the first computational quantification of WSS on placental intermediate villi at the end of the second trimester and, based on computational experiments, we infer the physiological WSS range in healthy pregnancy. Even in healthy placentas, there are regions within the placentone that are naturally devoid of villi (cavity) or exhibit reduced terminal villi formation (regions around the cavity). By overlaying spatial information on villi development from the literature and WSS estimates from simulations, we identify a WSS criterion for terminal villi underdevelopment and villi deficiency. We also examine how SA remodeling can alter the WSS acting on the placental villi surface.

Our simulation results show that incomplete SA remodeling can significantly alter hemodynamics within the placentone, leading to higher IVS velocities (> 6 mm/*s*), particularly around the free-of-villi cavity, which in turn increases WSS on placental villi. This elevated WSS may affect the structure and development of the placental villi and even lead to loss of villous tissue, as it can far exceed the WSS observed in a healthy placentone. Consistent with this, Fig 12b shows that less SA remodeling is associated with a greater extent of high IVS velocity and high WSS regions (orange), with these extending beyond the cavity. This pattern aligns with the reported formation of ECLs in some complicated pregnancies [44]. In severely unremodeled cases (25% and 0% SA remodeling), the maximum velocity at the top of the cavity generates a mean WSS exceeding 40 *Pa*. This WSS level is more than 26 times the maximum mean WSS at the top of the cavity when the SA is fully remodeled. Although no experimental data are available regarding the tolerance of syncytiotrophoblasts to sustained exposure to 40 *Pa* or higher, experiments on arterial endothelial cells have demonstrated that exposure to WSS in the range of 16.67−100Pa can be detrimental, leading to cell detachment even over a short period of time [28]. Based on this evidence and considering the delicate nature of syncytiotrophoblast cells [60,61], it is likely that incomplete SA remodeling may generate hemodynamic forces that can damage the placental microstructure and contribute to ECL development (Fig 12b). These findings are consistent with an *in vivo* imaging case report using four-dimensional high-definition flow Doppler ultrasound, in which a large SA jet was observed entering a placental lake and producing swirling flow in a case of fetal growth restriction [62]. We also observe higher blood velocities and, therefore, higher WSS near the veins (Fig 5). These results align with histological reports, which show sparser villi around the veins [2,7]. It is worth noting that because our macroscale model employs a static porous medium with uniform and constant porosity and permeability, it may underestimate the IVS velocity within the lobule, particularly in cases of severe partial SA remodeling. Physiologically, areas that become devoid of villi or have lower villi density due to exposure to higher IVS velocities would impose less flow resistance, further increasing IVS velocities deeper into the placentone.

Across all simulations, IVS velocity was the primary determinant of WSS. Changes in villous type (mature vs. immature intermediate villi) did not have a significant impact on WSS area distribution and on mean WSS (*p* > 0.05). This is likely due to the similar local permeability observed in the different configurations tested (Fig 10). Also, changing the villi orientation relative to the flow direction did not substantially affect the mean WSS compared with IVS velocity. However, the effect of villi orientation on mean WSS can be significant for a given IVS velocity, and villi orientation can considerably influence the WSS area distribution. The fact that larger orientation angles induced higher maximum WSS values (Fig 9) can be explained by the reduced local permeability, caused by greater misalignment between the villus axis and the flow.

Our simulation results indicate that a Newtonian blood model underestimates WSS on placental villi at low velocities. At the low shear rates encountered within microscale pores, red blood cells can reversibly aggregate, causing the apparent viscosity to increase [63]. This microscale phenomenon is not captured by the constant viscosity assumption of a Newtonian model. A non-Newtonian model is therefore recommended when IVS velocities fall below 15 mm/*s*, particularly in healthy placentones with fully remodeled SAs. Experimental studies have shown that even within a very low shear stress range, such as 0.0001−0.01Pa, syncytiotrophoblast responses vary with the applied shear stress [25]; thus, the choice of rheological model, Newtonian vs. non-Newtonian, can significantly influence predictions of cell behavior.

The WSS map obtained for a healthy placentone indicates that most placental villi are exposed to a mean WSS of 0.001−1Pa. This WSS map is consistent with previous experimental studies that subjected trophoblast cells to similar WSS values and observed beneficial effects on trophoblast responses [23,25]. For example, exposure of syncytiotrophoblast cells to WSS of 0.1 *Pa* increased the production of placental growth factors more than twofold compared to static conditions [23]. Another study reported that WSS within the range of 0.0001−0.01Pa promoted the formation of microvilli, which are necessary for improved feto-maternal biochemical exchange [25]. Around the free-of-villi cavity, WSS can rise up to 1.51 *Pa* (6 mm/*s*), which may explain terminal villi deficiency consistently reported in this region [2]. The biological response to WSS is likely dependent on both its magnitude and duration; physiological levels of WSS may act as mechanical cues involved in villous adaptation and proper development, whereas sustained exposure to much higher WSS may suppress villous formation, alter villous structure, or contribute to tissue injury. Together, these observations suggest that WSS provides a mechanobiological cue that helps shape villous development, and that deviations from the healthy range may hinder terminal villus formation or, in severe cases, lead to villous tissue loss.

Our model’s predicted WSS magnitude is also consistent with previous computational estimates of villous shear stress. For an IVS velocity of (1 mm/*s*), our model predicted a mean WSS between 0.29 and 0.33 *Pa*, depending on villous type and orientation, which is comparable to the previously reported WSS value of 0.23 *Pa* [33]. This difference may be due to differences in gestational age, model geometry, and the fact that our estimate is based on the aggregate result of 25 microscale samples rather than a single sample.

The present study provides a physiologically grounded and computationally feasible framework for estimating IVS velocity and villous WSS at the end of the second trimester. While explicitly resolving the complete placental microstructure in the full placentone model can be computationally intractable, simulating all 25 microscale samples required less than 40 CPU core-hours in total. Once a macroscale simulation is completed, microscale simulations can be performed independently for any location of interest and are therefore readily parallelizable. Thus, the computational cost scales approximately with the number of microscale locations evaluated rather than with the total number of villi within the placentone. Furthermore, the proposed strategy of coupling macroscale and microscale models may also be applicable to other multiscale biological problems involving flow through complex heterogeneous tissues in which the microscale structure cannot be feasibly resolved throughout the entire computational domain.

Although representative parameters were used in this study to define the model geometry and inflow conditions, the framework can incorporate patient-specific variability in placentone size, villous density, SA geometry, degree of remodeling, and inlet flow conditions if clinical measurements are available. The WSS estimates generated by this framework can serve as a provisional computational benchmark to guide future microfluidic experimental verification of cultured trophoblast cell responses to physiologically and pathologically relevant WSS levels. Throphoblast responses to different WSS levels could be assessed by measuring the secretion of signaling proteins involved in placental villous development [12,23]. Following experimental and clinical validation, these computational estimates could provide a basis for integrating clinically obtainable measurements of uteroplacental physiology and placental anatomy with computational modeling to infer the local mechanical environment experienced by developing placental villi.

## Limitations

In this study, our primary focus was to quantify WSS on the placental microstructure and identify a WSS range that can potentially hinder the development of the terminal villi. For this purpose, we based our analysis on a healthy placentone by quantifying WSS in areas naturally devoid of terminal villi. The flow in our simulations was then assumed to be steady, which is a reasonable assumption under healthy conditions [8]. However, in partially remodeled SA cases, where flow pulsatility may not be negligible [2,64], the assumption of a steady flow would fail to capture WSS oscillations. Furthermore, the fluid-villi interactions can cause the IVS pore diameters to cyclically increase and decrease due to pulsatile flow, thereby affecting local velocity and, in turn, WSS. Beyond WSS levels, WSS oscillations may also be important because villous surfaces would be exposed to repeated variations in WSS rather than a steady-state WSS condition, potentially affecting mechanotransduction and cellular signaling.

The use of a porous medium model with constant porosity and permeability may underestimate IVS velocities in cases of incomplete SA remodeling and, consequently, may underestimate villous WSS, particularly in regions deeper within the placentone. In severely unremodeled SA cases, the incoming high-momentum blood flow jet can carve channels within the villi-containing region to exit the lobule through less-resistant paths [2], resulting in increased IVS velocities and greater flow heterogeneity due to local changes in porosity and permeability that are not captured by a constant porosity and permeability model. In addition, loss of villous tissue in ECLs can also contribute to increased IVS velocities. Therefore, dynamic porosity and permeability models may be required to more accurately capture IVS flow patterns in cases of incomplete SA remodeling.

In our simulations, we considered the SA as a straight arterial segment. Although coiling is reduced during gestation [65], and IVS velocities are determined mainly by the velocities at SA opening, SA residual coiling can result in an asymmetric velocity profile at SA opening and, therefore, an asymmetric IVS velocity field. This effect should be further investigated in future work.

Overall, several sources of uncertainty and biological variability should be considered when interpreting the predicted WSS values. Microstructural variability was explicitly incorporated through the stochastic generation of microscale villous geometries based on experimentally reported IVS pore-size distributions; aggregate WSS estimates converged with 25 independently generated samples. We further evaluated the variability associated with villous type and orientation. At the macroscale, however, several geometric and physiological parameters were defined using representative literature values in the absence of subject-specific measurements at the end of the second trimester. These include placentone dimensions, cavity geometry, porosity, permeability, and SA geometry. Consequently, the WSS values and proposed thresholds should be interpreted as physiologically grounded estimates rather than patient-specific predictions. Future studies incorporating subject-specific imaging and physiological measurements will enable systematic propagation of these sources of variability through the multiscale framework and quantification of their contribution to uncertainty in predicted WSS values.

## Conclusion

In this study, we established a novel multiscale approach to estimate WSS on placental villi in a computationally efficient way. Our simulation results indicate that a Newtonian blood-flow assumption cannot accurately estimate WSS on placental villi, particularly in a healthy placentone. We also show that incomplete SA remodeling can induce hemodynamic conditions that promote ECL formation, consistent with clinical and histopathological observations. Based on the IVS velocities and corresponding villous WSS around the free-of-villi cavity in a healthy placentone, we propose that a mean WSS within the range of 0.71−1.44Pa can potentially hinder the formation of terminal villi. These WSS estimates can inform future experimental studies on trophoblast mechanobiology.

From a clinical perspective, these findings suggest that impaired SA remodeling may alter uteroplacental blood flow, changing the local hemodynamic environment and the shear stresses experienced by placental villi. Collectively, the results provide a potential mechanistic link between abnormal uteroplacental hemodynamics and adverse pregnancy outcomes, including fetal growth restriction and preeclampsia.
